# Current Relative Value Scale Methodology Underestimates Perioperative Workload in Hip Arthroscopy

**DOI:** 10.1016/j.asmr.2025.101157

**Published:** 2025-04-25

**Authors:** Shelby C. Hodges, Juan J. Gordillo, Clay A. Rahaman, Mathew Hargreaves, Maxwell L. Harrell, Dev Dayal, Thomas B. Evely, Eugene W. Brabston, Amit M. Momaya, Aaron J. Casp

**Affiliations:** a Heersink School of Medicine, University of Alabama at Birmingham, Birmingham, Alabama, U.S.A.; b Department of Orthopaedic Surgery, University of Alabama at Birmingham, Birmingham, Alabama, U.S.A.

## Abstract

**Purpose:**

To evaluate the accuracy of the current times and work relative value units (wRVUs) recommended by the Relative Value Scale (RVS) Update Committee (RUC) for the perioperative work associated with hip arthroscopy in a single surgeon’s practice.

**Methods:**

The RUC was contacted to obtain a list of perioperative tasks and times allotted for these tasks for hip arthroscopy procedures (Current Procedural Terminology codes 29914, 29915, and 29916). A board-certified, sports medicine fellowship–trained orthopaedic surgeon recorded the time it took to perform each perioperative task. Recorded times were multiplied by their respective Centers for Medicare & Medicaid Services–assigned intensity coefficients to calculate the wRVUs for preservice and postservice tasks. Calculated and allotted wRVUs were compared for accuracy.

**Results:**

The tasks timed in this study were allotted 83 minutes by the RUC with a wRVU of 1.72. Our study found that these same tasks significantly differed, at 93.4 minutes and total wRVU of 1.95 (*P* < .001). The overall time it took to perform perioperative tasks for hip arthroscopy was underestimated by 10.4 minutes, resulting in an undervaluation of wRVU by 0.23.

**Conclusions:**

In a single surgeon’s practice, the RUC underestimates the time required to perform perioperative tasks for hip arthroscopy procedures.

**Clinical Relevance:**

It is important to determine whether the amount surgeons are reimbursed for certain procedures, such as hip arthroscopy, is an accurate reflection of actual cost. Discrepancies between reimbursement and actual costs can influence the financial viability of offering such procedures, impacting access to care, procedural volume, and potentially long-term outcomes, particularly as health care systems move toward value-based reimbursement models.

Since the Centers for Medicare & Medicaid Services (CMS) was created in 1965, physicians began receiving payments based on usual, customary, and reasonable charges.^1^^,^^2^ This fee-for-service method reimbursed physicians based on what they would normally charge patients for the particular service provided in combination with general charge patterns in the community.^3^ The CMS then decided to design a fixed payment system based on the premise that the previous reimbursement system rewarded increased provider spending and prompted unnecessary surgical procedures as opposed to adequate medical interventions.^2^, ^3^, ^4^

Four aspects were identified to define physician work: time, technical skill and physical effort, mental effort and judgment, and psychological stress.^5^, ^6^, ^7^ For conciseness, the CMS consolidated these into 2 variables: time and intensity.^7^ The team that created and oversees this system is the Relative Value Scale (RVS) Update Committee (RUC). Through surveys sent to select practicing physicians, the RUC created the RVS to summarize all diagnostic codes and values assigned to each code.^6^^,^^7^ The RVS assigns each procedure a relative value unit (RVU) based on expense and work required and reimburses physicians a fixed rate depending on the RVU.^2^^,^^5^

Despite the well-intended purposes of creating this system, it is embedded with flaws that demand attention and modification. The surveys administered by the RUC have had low and unreliable response rates, ranging from 55.9% to 69% per specialty, requiring only 30 responses from practicing physicians.^8^^,^^9^ Additionally, nearly 90% of recommendations in the surveys are accepted by CMS despite minimal research into the accuracy of the values.^10^, ^11^, ^12^ Many procedures often go 5 to 10 years without re-evaluations and updates from the RUC.^12^

Prior studies have shown discrepancies in RUC estimated times and work relative value units (wRVUs) for procedures based on survey data as opposed to directly timing perioperative tasks.^12^, ^13^, ^14^, ^15^, ^16^, ^17^, ^18^ The purpose of this study was to evaluate the accuracy of the current times and wRVUs recommended by the RUC for the perioperative work associated with hip arthroscopy in a single surgeon’s practice. We hypothesized that the RUC would underestimate the amount of time it takes to perform the arthroscopically aided hip procedures with Current Procedural Terminology codes 29914, 29915, and 29916.

## Methods

This study was approved by an institutional review board under exempt category 4. The RUC was contacted to obtain a list of perioperative tasks and times assigned to those tasks for arthroscopically aided hip procedures based on Current Procedural Terminology codes 29914, 29915, and 29916. Specifically, code 29914 indicates femoroplasty, code 29915 indicates acetabuloplasty, and code 29916 indicates labral repair. These codes were chosen because they are believed to require similar technical skills and experience to perform, especially in the realm of perioperative work. Among the list of tasks provided by the CMS, only perioperative preservice and postservice tasks were included in this study. Intraservice work, comprising the tasks performed from the first skin incision until the final skin closure, was excluded owing to its inherent variability. Different hip pathologies require varying degrees of surgical intervention, and intraoperative times are highly dependent on surgeon experience and career stage. Given that this study was conducted at a single institution where only 1 surgeon performs hip arthroscopy, including intraservice work would not provide a representative spread of surgical complexity and efficiency. Instead, we focused on perioperative work, which is more consistent across surgeons and institutions.

Thirty-two hip arthroscopy procedures were timed over a 3-month period. All surgical procedures included were performed by 1 board-certified, sports medicine fellowship–trained orthopaedic surgeon (A.J.C.) at a single hospital.

In calculating total perioperative work, the CMS subdivides preservice and postservice work into time- and group-specific subsections. Preservice work is performed before the procedure and is subdivided into 3 categories: (1) pre-evaluation; (2) pre-positioning; and (3) pre-scrub, dress, and wait time. Postservice work is completed after the procedure and includes all the tasks performed on the same day as the procedure and within the hospital. Tasks performed outside and inside the operating room (OR) were prospectively timed as the events occurred by the attending physician. Individual perioperative tasks specified by the RUC in the preservice and postservice periods were timed for consecutive hip arthroscopies. A list of timed tasks is available in Table 1.

**Table 1:** Components of Preservice and Postservice Tasks

Task	Mean Time, min
Pre-evaluation	48.4 ± 5.2
Write preadmission orders for preoperative medications	1.8 ± 0.6
Review results of preadmission testing	4.8 ± 2.0
Update H&P	2.6 ± 0.5
Meet with patient/family to review plan	3.8 ± 1.0
Review and update consent, verify operative supplies, and monitor and assist with arranging of patient on fracture table in lateral position	35.4 ± 3.0
Pre-positioning	10.6 ± 1.7
Indicate areas of skin to be prepared and mark surgical incisions	1.0 ± 1.0
Assess position of extremities and head, with adjustment if needed	1.2 ± 0.4
Perform preparation and draping of leg	7.2 ± 1.3
Perform padding of bony prominences and pelvic post	1.2 ± 0.5
Scrub, dress, and wait time	10.0 ± 2.3
Perform surgical “timeout” with OR surgical team	1.0 ± 0.0
Perform scrubbing and gowning	2.2 ± 0.7
Assess access to joint with fluoroscope	6.8 ± 2.0
Postservice	24.2 ± 3.1
Perform application of dressing and sling	2.5 ± 1.1
Monitor patient during reversal of anesthesia	3.0 ± 1.0
Perform transfer of patient from OR to gurney	1.5 ± 0.6
Perform transfer of patient from OR to PACU	2.0 ± 0.8
Communicate with health care professionals	1.8 ± 0.6
Discuss procedure and outcome with family	4.7 ± 0.9
Write brief operative note or complete final operative note and place in chart	2.5 ± 0.5
Dictate operative report and copy referring physician(s)	5.2 ± 1.0
Assess circulation, sensation, and motor function of operated extremity	1.0 ± 0.4
Total average perioperative time	93.4 ± 7.3

NOTE. Data are expressed as mean ± standard deviation.

H&P, history and physical examination; OR, operating room; PACU, postanesthesia care unit.

The attending surgeon in this study worked with a single postgraduate year-2 resident who rotated services every 8 weeks. The perioperative setup involved the circulating nurse, the attending surgeon, and the resident. The attending surgeon and resident were involved in patient transfer and were responsible for postoperative orders. The surgeon has been in practice for 4 years, with OR times initially decreasing as efficiency improved. At the time of this study, OR workflow had reached a steady-state plateau.

The CMS assigns an intensity coefficient to each of the aforementioned categories. Times collected for each task were averaged and added together to assign a mean time and standard deviation (SD) to each preservice and postservice task. The wRVU was calculated from the product of the CMS-assigned intensity coefficient and the mean procedural time. One-sample *t* tests were performed to assess for differences between the RUC standard and collected study data for each task’s mean time and wRVU correlate.

## Results

Of the 32 patients recorded, 15 (47%) were men and 17 (53%) were women. The average age of our study population was 35.3 years (SD, 15.7 years). The mean pre-evaluation time was 48.4 minutes (SD, 5.2 minutes); pre-positioning time, 10.6 minutes (SD, 1.7 minutes); scrub, dress, and wait time, 10.0 minutes (SD, 2.3 minutes); and postservice task time, 24.2 minutes (SD, 3.1 minutes). Mean times of subtasks are displayed in Table 1.

As shown in Table 2, mean time and wRVUs for preservice tasks were significantly greater than the RUC standards by 6.0 minutes and 0.135 wRVUs (*P* < .001). Under preservice tasks, mean time and wRVUs were significantly greater for pre-evaluation tasks by 15.4 minutes and 0.345 wRVUs (*P* < .001) and mean time and wRVUs were significantly less for pre-positioning by 9.4 minutes and 0.211 wRVUs (*P* < .001). Mean time and wRVUs for postservice tasks were significantly greater by 4.2 minutes and 0.990 wRVUs (*P* < .0001). Overall, the time it took to perform perioperative tasks for hip arthroscopy was underestimated by 10.4 minutes, resulting in an undervaluation of 0.23 wRVUs.

**Table 2:** Comparison of wRVUs: RUC Versus Study Data

Task	Intensity Coefficient	RUC Standard		Study Data∗ (N = 32)	
		Mean Time, min	wRVU	Mean Time, min	wRVU
Preservice		63	1.268	69.0 ± 5.8†	1.403 ± 0.13
Pre-evaluation	0.0224	33	0.739	48.4 ± 5.2†	1.084 ± 0.12
Pre-positioning	0.0224	20	0.448	10.6 ± 1.7†	0.237 ± 0.04
Pre-scrub, dress, and wait time	0.0081	10	0.081	10.0 ± 2.3	0.081 ± 0.02
Postservice	0.0224	20	0.448	24.2 ± 3.1†	0.547 ± 0.07
Total		83	1.716	93.4 ± 7.3†	1.944 ± 0.17

RUC, Relative Value Scale (RVS) Update Committee; wRVU, work relative value unit.

∗ Data are expressed as mean ± standard deviation.

† *P* < .001.

## Discussion

The primary finding of this study suggests that the current wRVU system established by the RUC and CMS does not effectively measure the perioperative workload associated with hip arthroscopy procedures. This study found that the RUC underestimates perioperative work time in hip arthroscopy procedures by 10.4 minutes and wRVU by 0.23, consisting of a 13.37% increase when compared with the current CMS standards.

The results from this study support conclusions from similar studies measuring wRVU accuracy in orthopaedic surgery.^14^^,^^19^ One study found that 14.5 minutes are unaccounted for in the perioperative period for shoulder arthroplasty procedures.^14^ The discrepancy in reimbursement is further highlighted when comparing RVUs for primary versus revision procedures. A retrospective study comparing recorded operative times and RVUs for revision versus primary total knee arthroplasty (TKA) procedures from the National Surgical Quality Improvement Program database found that current RVU allocations do not reflect the added complexity, time commitment, and additional effort required during the preoperative planning and postoperative care of patients.^17^ Another study using the same database found that RVU-per-minute reimbursement rates were nearly identical for primary and revision shoulder arthroplasty cases, despite the additional complexity of revision procedures.^13^ This finding was further supported in a 2021 retrospective review of revision versus primary TKA procedures performed at a single urban academic medical center, determining that as complexity increases, physicians face less reimbursement per minute and per RVU.^16^

Furthermore, as observed in this study, several prospective studies found that preoperative work is significantly undervalued. One study found that there were nearly 2 hours of work unaccounted for in total hip arthroplasty and TKA procedures during the preoperative period, and another study found that the preservice wRVU of these same procedures is underestimated by 0.285.^15^^,^^18^ Collectively, these studies show that physician workload has had a consistent upward trajectory over time while RVUs have stayed relatively static. A retrospective study by Kufta et al.^20^ evaluated the changes in reimbursement for the 7 most common hip arthroscopy procedures. Even though the reimbursement rate marginally increased by 1.8% from 2011 to 2021, when adjusting for the increase in the consumer price index of 29.0%, the adjusted reimbursement rate decreased by 21.1%. Unlike the study by Kufta et al., which examined reimbursement rate trends from 2011 to 2021, our study quantifies the underestimation of perioperative workload within a single surgeon’s practice. By providing a real-time assessment of workload discrepancies, our findings highlight the need for reimbursement adjustments that better reflect the true demands of hip arthroscopy perioperative work.

Increased documentation time may also be contributing to this discrepancy. Although the implementation of electronic medical record (EMR) software allows physicians to access information about a patient in real time, it has also created a system whereby physician time and focus shift from interacting with patients toward retrieving information for the EMR. In 2008, one retrospective study found an 8.5% decrease in time devoted to patient care relative to the total time spent on patient consultations before and after the implementation of EMR software.^21^ Two prospective studies found a nearly 9% increase in time spent on documentation after the implementation of EMR software.^22^ Specifically, time spent reviewing preoperative documentation more than doubled from 7.4% to 15.9% after the implementation of EMR software.^21^ Although there is an incentive to continue using the latest technological advances, these changes contribute to the overall increase in time spent during the perioperative period and, therefore, should be considered by the RUC when evaluating procedure time and intensity.

Perioperative work to address risk factors may cause increased time associated with the patient that has not been accounted for by the RUC.^23^, ^24^, ^25^ As further research hones in on the patient factors associated with negative outcomes after surgery, additional time may be needed that is not currently accounted for.^26^^,^^27^ Two Web-based surveys completed in 2020 by surgeons from the American Association of Hip and Knee Surgeons indicated an 86% to 87% increase in preoperative work burden since 2013.^28^ Educating the patient on postoperative regimens such as nutrition, diet, and exercise regimens has been shown to be effective in reducing the postoperative length of stay and the number of postoperative complications.^23^^,^^25^ Although these advancements in research improve patient care, they also lengthen the time spent in the perioperative period, a factor not considered by the CMS.

The expansion of knowledge regarding surgical approaches alters the time and intensity required for each procedure. A retrospective study conducted in 2017 found that the number of hip arthroscopies performed in the United States has risen over time with a correlated increased complexity and diversity of procedures performed.^29^ Studies advocating for surgical intervention in younger patients before the development of joint space narrowing have shown a rise in the number of cases, especially in patients younger than 50 years, adding to the complexity of the procedure.^29^, ^30^, ^31^ Additionally, studies have indicated an upward trend in the use of surgical techniques requiring a higher level of skill as advancing literature has shown more favorable outcomes using these techniques.^29^ For example, the use of labral preservation as opposed to debridement for the prevention of the progression of arthritis in younger patients has occurred at a higher rate over time despite its increased skill requirement.^3^^,^^32^

Failure of the CMS and RUC to consider additional EMR documentation and the increased technical difficulty that comes with further research in the hip preservation field will continue to widen the gap between perioperative time spent and the intensity of work performed by physicians and their reimbursement. Re-evaluating wRVUs more frequently and incorporating prospective data collected by physicians may mitigate the size of the discrepancy seen and more accurately recognize the increased perioperative work required by physicians for hip arthroscopy procedures. Future studies may encompass a societal or multi-institutional design that would bring forth generalizable results that would allow for public health–level discussions on reimbursement and wRVUs.

### Limitations

This study is not without limitations. This study focuses on a single academic hospital system, and data were collected for 1 surgeon. Therefore, the data may not be extrapolated to other surgeons or other types of surgical settings such as surgery centers. Other variables that may affect perioperative task times and data extrapolation are inclusion of resident physicians or physician extenders, rotation of the surgical team, maturity of the surgeon’s practice in hip arthroscopy, and trends in operative time. In addition, the type of EMR system used, EMR requirements, and hospital protocols may vary between hospitals and may affect the results. Moreover, there is a potential for timing errors despite capturing times prospectively. However, the margin of error for times collected prospectively is less than the estimated time collected retrospectively via survey data. The retrospective survey data collected by the RUC also had low and unreliable response rates that may have resulted in an inaccurate estimation of surgeon workload, ultimately influencing the study’s calculated differences with the prospectively collected data.

## Conclusions

In a single surgeon’s practice, the RUC underestimates the time required to perform perioperative tasks for hip arthroscopy procedures.

## Disclosures

The authors declare the following financial interests/personal relationships which may be considered as potential competing interests: E.W.B. reports board membership with EBSCO and reports a consulting or advisory relationship with Link Orthopaedics and Orthopaedic Design. A.M.M. reports a consulting or advisory relationship with Arthrex, ConMed Linvatec, Fidia Pharma USA, and Miach Orthopaedics; reports board membership with *Arthroscopy*; and owns equity or stocks in Reparel. A.J.C. reports a consulting or advisory relationship with Arthrex and reports board membership with American Orthopaedic Society for Sports Medicine. All other authors (S.C.H., J.J.G., C.A.R., M.H., M.L.H., D.D., T.B.E.) declare that they have no known competing financial interests or personal relationships that could have appeared to influence the work reported in this paper.
